# Application of long-read sequencing to elucidate complex pharmacogenomic regions: a proof of principle

**DOI:** 10.1038/s41397-021-00259-z

**Published:** 2021-11-05

**Authors:** Maaike van der Lee, William J. Rowell, Roberta Menafra, Henk-Jan Guchelaar, Jesse J. Swen, Seyed Yahya Anvar

**Affiliations:** 1 Department of Clinical Pharmacy and Toxicology, Leiden University Medical Center, Leiden, the Netherlands; 2 Leiden Network of Personalized Therapeutics, Leiden, the Netherlands; 3 Pacific Biosciences, Menlo Park, CA USA; 4 Department of Human Genetics, Leiden University Medical Center, Leiden, the Netherlands; 5 Present Address: OKRA Technologies, Cambridge, UK

**Keywords:** Genetics research, Sequencing

## Abstract

The use of pharmacogenomics in clinical practice is becoming standard of care. However, due to the complex genetic makeup of pharmacogenes, not all genetic variation is currently accounted for. Here, we show the utility of long-read sequencing to resolve complex pharmacogenes by analyzing a well-characterised sample. This data consists of long reads that were processed to resolve phased haploblocks. 73% of pharmacogenes were fully covered in one phased haploblock, including 9/15 genes that are 100% complex. Variant calling accuracy in the pharmacogenes was high, with 99.8% recall and 100% precision for SNVs and 98.7% precision and 98.0% recall for Indels. For the majority of gene-drug interactions in the DPWG and CPIC guidelines, the associated genes could be fully resolved (62% and 63% respectively). Together, these findings suggest that long-read sequencing data offers promising opportunities in elucidating complex pharmacogenes and haplotype phasing while maintaining accurate variant calling.

## Introduction

Pharmacogenomics (PGx) is crucial for individualizing drug dosages and thereby improving drug therapy outcomes [1, 2]. PGx relies on inferred phenotypes based on known variants in pharmacogenes. Nonetheless, not all genetic variability in drug response and enzyme activity can be explained by routine PGx genetic assays [3, 4], due to several factors. First, current genotyping assays are unable to fully resolve the genetic makeup of all genes involved in drug response [5–7]. Second, the mechanism of action of a drug and/or its metabolic pathway is not always fully understood [4, 8]. It is essential to be able to explain all genetic components driving variable drug response in order to assess what part of variability is genetic and what part can be explained by other factors. This is, however, challenged as the majority of pharmacogenes are at least in part located in complex genomic regions or contain variants like tandem-repeats and pseudogene hybrid conformations [9]. Currently applied genotyping technologies are based either on SNV (Single Nucleotide Variant) microarrays or short-read sequencing [10, 11]. Both approaches are limited in characterizing these complex regions [12–15], as they fail to adequately and reliably resolve highly homologous regions and identify PGx variants [7, 16, 17]. Moreover, with haplotype phasing it could be determined if variants are located on the same allele or if they are on different alleles, potentially leading to differences in phenotype assignment. Currently, PGx diplotypes are phased based on linkage disequilibrium. While this results in accurate haplotypes on a population scale it does not always result in accurate assumptions on an individual level. The impact of these challenges in clinical practice is high [5]. For example, the complex gene *CYP2D6*, is involved in the metabolism of 20–30% of commonly prescribed drugs [18] and cannot be fully characterized by short-read sequencing.

In recent years the long-read sequencing technologies from Oxford Nanopore and PacBio have shown to be capable of characterizing complex (pharmaco)genomic regions [19–21]. For these regions, long and high-quality reads significantly improve variant calling precision and allow for resolution of fully phased diplotypes.

The value of long-read sequencing for disease diagnostic purposes has previously been illustrated [7, 16, 22–26]. PacBio sequencing has been shown capable of characterising *CYP2D6*, by covering the entire gene locus in one high-quality long read [7, 16, 26–29]. More recently, long-read sequencing has also been applied for the HLA genes in relation to PGx [29, 30]. In addition, its application has been used in numerous challenging clinical diagnostic research assays such as long tandem repeat in *FMR1* gene linked to Fragile X syndrome [22] and in resolving the *PKD1* gene to detect mutations associated with polycystic kidney disease [23]. Finally, long-read sequencing facilitates haplotype phasing without the need for computational approaches and/or pedigree information. This can be of crucial importance in PGx leading to more accurate phenotype predictions [15]. The combination of PGx complexity and haplotype phasing indicates that long-read sequencing has the potential to substantially improve our ability to correctly predict drug metabolizer phenotypes. In this proof-of-concept paper, we assess the potential of long-read PacBio sequencing to resolve complex PGx regions by using available sequencing data of the well-characterised Genome in a Bottle (GIAB) reference sample HG002.

## Results

### Data description

**Fig. 1:**
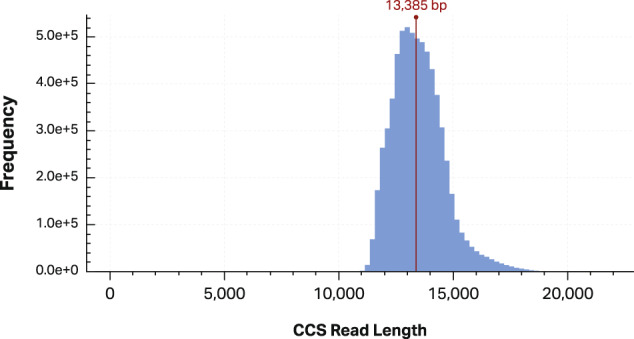
Read length distribution. Distribution of read length of genome in a bottle sample HG002 after sequencing on Pacific Bioscience sequel platform and construction of circular consensus sequence.

Previously published sequencing data of the well-characterized HG002 GIAB sample were obtained [19]. This data consists of 6,728,123 reads with a median length of 13.4 kb, covering 97.5% of the genome (Fig. 1) with an average mapped coverage of 28-fold. Approximately 5 million genetic variants were detected using GATK (Genome Analysis Toolkit) HaplotypeCaller [31] and DeepVariant [32].

### High precision and recall in variant calling

For the 100 selected pharmacogenes, precision and recall compared to the benchmark truth set GIAB v3.3 was determined. For SNVs, GATK HaplotypeCaller and DeepVariant achieved similar precision and recall above 99.8% (Table 1). However, the DeepVariant caller achieved a much better performance in detecting indels (>98%) compared to GATK (precision: 94.5% and recall: 86.1%). When comparing to the genome wide results reported by Wenger et al, the precision and recall in detecting variants in the pharmacogenes are superior [19]. When stratifying results on complex regions (Table S2), accuracy remained high, with recall and precision >95% for all regions for both indels and SNVs. For the GATK caller, the accuracies were lower, (85-100% compared to 97-100% for DeepVariant caller). The drop in accuracy could be attributed to lower performance for tandem repeats and homopolymers (Table S2 and Fig. S1).

**Table 1:** Variant calling performance for pharmacogenes.

Variant caller	SNVs			Indels		
	Precision (%)	Recall (%)	F1 (%)	Precision (%)	Recall (%)	F1 (%)
GATK haplotype caller	99.88	99.96	99.92	94.47	86.12	90.10
DeepVariant (CCS model)	99.84	100.0	99.92	98.74	98.00	98.37

Measured against the Genome in a Bottle benchmark v.3.3.2. using both GATK variant caller and DeepVariant. *SNV* single nucleotide variant, *Indels* insertions and deletions, *GATK* genomic analysis toolkit, *CCS* circular consensus sequence.

To assess the accuracy of SV calling in pharmacogenes, SV calls were compared with the SV benchmark set for all SVs over 50 bp. However, the high confidence GIAB regions did not cover all 100 genes. 46 genes were excluded, 12 genes were partially and 42 were fully overlapping with the GIAB curated data (Table S3). In total, 22 SVs (>50 bp) were identified in the 54 pharmacogenes compared to 23 catalogued in the benchmark set (Table S4). Two calls were regarded as false negative and one call as false positive. Together, assessing the performance of detecting SVs in PGx regions resulted in recall of 91.3% and precision of 94.5%. The high recall and precision in pharmacogenes suggest that there is no loss of accuracy with the use of long-read sequencing data compared to current benchmarks, whilst improving the detection of complex genetic variants.

### Haplotype phasing and haploblocks

**Fig. 2:**
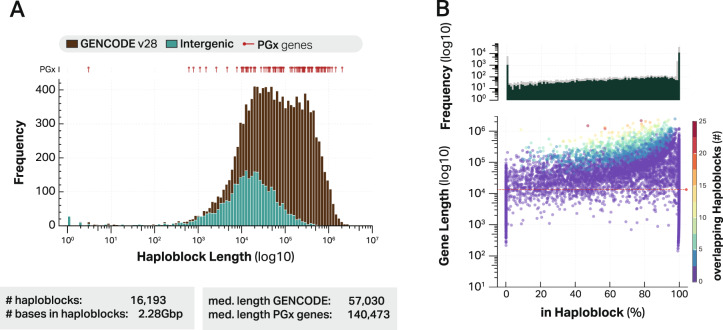
Haploblock resolution of GENCODE features. **A** haploblock length distribution stratified by Gencode features and intergenic regions, overlap with pharmacogenes is highlighted in red. **B** For each protein coding feature the percentage that were resolved into haploblocks compared to the feature length. The red line reflects the mean read length. The majority of haploblocks are larger than the mean read length, indicating that not read length but the number of heterozygous variants is decisive for the length of a haploblock.

Using WhatsHap [33], reads were phased and resolved into haploblocks based on all identified variants. Each haploblock describes one stretch of fully phased sequence allowing for a complete characterisation of that region, representing a maternal or paternal allele. Notably, 71.2% of the genome could be phased into 16,193 haploblocks with a total haploblock length of 2.3 billion base pairs and a median haploblock size of 40,302 bp (range: 1–2.9 million bp). A clear distinction in haploblock size was observed between intergenic regions (median of 14,960 bp) and Gencode features (median of 56,743 bp), Fig. 2A. The vast majority of Gencode features was fully phased into haploblocks (Fig. S2 and S3). In particular, 71% of all protein coding features could be completely phased (≥90%) and an additional 22% were partially phased while 7% remained unresolved (≤10% phased). Similar patterns were observed for other Gencode features (Figs. S2 and S3). Read length does not seem to be the main limiting factor in resolving haplotypes as the percentage of a feature covered in haploblocks is independent of feature length (Fig. 2B). In addition, the majority of haploblocks (57.7%) exceed the median read length, indicating that not read length but the number of heterozygous variants and number of reads aligned to a given genomic region are the limiting factors in haploblock construction.

### Pharmacogenes

**Fig. 3:**
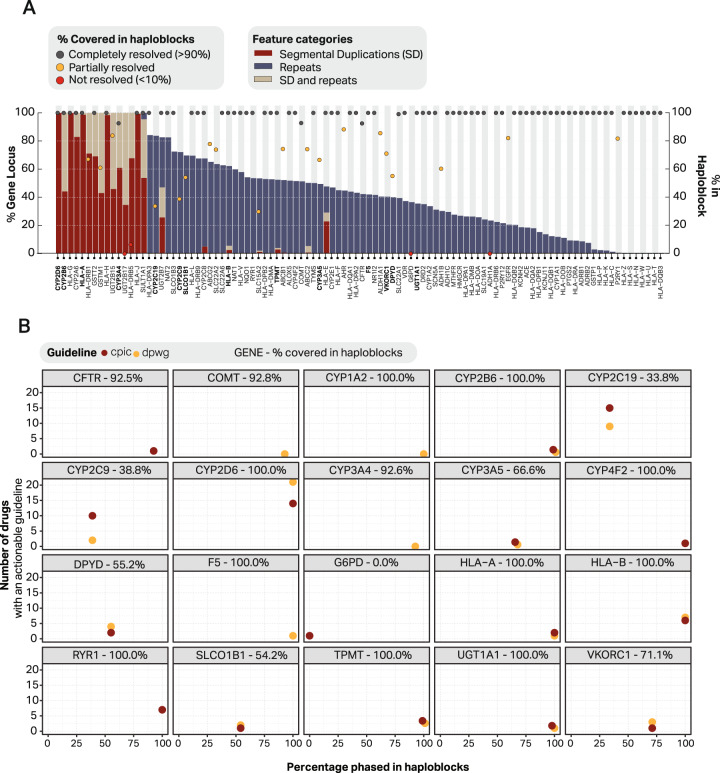
Complexity of pharmacogenes and proportion solved in haploblocks. In (**A**), the pharmacogenes and their complexity related to the percentage covered in haploblocks. In bold genes included in the Ubiquitous pharmacogenomics (U-PGx) passport. **B** for genes included in the CPIC of DPWG guidelines the number of available actionable guidelines is mapped to the percentage of each gene which is phased into haploblocks. Actionable is defined as guidelines which recommends a dose change or drug switch. For each gene the percentage resolved in haploblocks is included in the panel headers. CPIC Clinical Pharmacogenetics Implementation Consortium, DPWG Dutch Pharmacogenetics Working group.

For each of the 100 selected pharmacogenes the portion of the genes located in a complex region was determined—with complex defined as genomic regions that overlap with segmental duplications (SD) or repeats. In total, 15 pharmacogenes were classified as 100% complex whereas eight pharmacogenes did not show any overlap with SDs or repeats (Fig. 3A).

For each of the 100 loci, almost all variants could be accurately called (precision and recall >99.8%). Subsequent phasing resulted in haploblocks with a median length of 140,473 bp, resulting in the majority (73/100) of the features being fully phased into haploblocks (Fig. 3A). Most significantly, of the 15 pharmacogenes classified as fully complex, 9 could be fully phased, 4 for at least 60% and the last two could not be phased. Of the notoriously complex HLA-genes, 35 out of 37 were fully resolved, the remaining two (*HLA-DRB5* and *HLA-DRB1*) were resolved for 6.4% and 67.1%, respectively.

Nonetheless, several important pharmacogenes could only be partially phased into haploblocks. For example, *G6PD*, *DPYD* and *CYP2C19* were resolved for 0%, 55% and 34%, respectively. As *G6PD* is located on the X chromosome and the individual sequenced is male, it is not possible to phase the locus into two alleles resulting in 0% of the locus being covered in phased haploblocks. For *DPYD* the cause lies in a combination of long gene length (~900,000 bp) and a low number of variants leading to large stretches without heterozygous variants resulting in broken haploblocks (Fig. S4). For *CYP2C19*, there is a large portion in the centre of the gene which is homozygous for all variants. More specifically, in the entire *CYP2C19* locus there are 52 variants of which 33 are homozygous, resulting in fragmented phased blocks (Fig. S4). Yet, as all regions have been sequenced, it is still possible to assign haplotypes and phenotypes using the current Dutch Pharmacogenetics Working Group (DPWG) and Clinical Pharmacogenetics Implementation Consortium (CPIC) guidelines and phasing assumptions.

To assess the clinical utility, diplotypes and phenotypes were assigned based on the variant panel from the Ubiquitous pharmacogenomics (U-PGx) consortium and a previously developed pipeline [15]. A total of 1,418 variants were identified in 10 key pharmacogenes included in the panel, from which, 38 variants were considered in the phenotyping panel (Table S5). Clinically relevant variants in the *CYP3A5*, *CYP2D6* and *VKORC1* genes were identified. For CYP3A5, the rs776746 (g.99672916 C > T) variant was found on both alleles resulting in a CYP3A5*3/*3 genotype and a Poor Metabolizer phenotype. For *CYP3A5* a PM status is regarded as not actionable due to this being the most common phenotype in Caucasians. For *CYP2D6* and *VKORC1* the inferred phenotype was divergent from the wildtype. In the *CYP2D6* locus, both the rs3892097 (g.42128945 C > T) and the rs1065852 (g.42130692 G > A) variant were found to be heterozygous. With phasing, it was determined that the variants were located on the same allele resulting in a CYP2D6*1/*4 diplotype and inferred CYP2D6 intermediate metabolizer (IM) phenotype. Moreover, given the presence of the non-functional *CYP2D7* pseudogene which shares >90% of its sequence with *CYP2D6*, it is of importance to exclude any interference of *CYP2D7* reads to accurately determine *CYP2D6* haplotypes [5]. The reads were sufficiently long to allow for a clear distinction between *CYP2D6* and *CYP2D7* without any ambiguously mapped reads (Fig. S4). The same was observed for *CYP2B6* and its pseudogene *CYP2B7P* and for the *CYP3A* locus of which all genes share high sequence homology (Fig. S4). For *VKORC1*, a homozygous variant (NC_000016.10:g.31093557 G > A) was identified, leading to the 1173TT genotype resulting in a decreased activity (Fig. S4). Overall, these results indicate that, according to publicly available consensus guidelines, this individual would require dose adjustments for drugs that are a substrate to CYP2D6 and VKORC1.

### Clinical relevance

In total, 15 genes included in this study are represented in the CPIC and/or DPWG guidelines, resulting in a total of 56 and 67 gene-drug interactions for the DPWG and CPIC guidelines respectively (Fig. 3, Table S6). Of these genes 10 (66.7%) were completely resolved in phased haploblocks. The genes which were fully resolved are involved in 35 of the gene-drug interactions in DPWG and for 35 gene-drug interactions in CPIC. For the remaining genes, variants could still be accurately identified, allowing for haplotype assignments according to current clinical practice which uses non-phased genetic data.

## Discussion

In this proof-of-concept study, we have shown that long-read sequencing yields high quality variant calls in all selected pharmacogenes. Compared to the genome-wide analysis [19], results for PGx genes are superior with regards to variant calling accuracy and resolution of larger phased haploblocks. In addition, the majority of the selected pharmacogenes could be fully resolved in phased haploblocks.

Based on variant calling alone, long-read whole genome data can be used for routine PGx similar to the way NGS is used [15, 34, 35]. Moreover, long-read sequencing offers the benefit of resolving paternal and maternal alleles. Given the polymorphic nature of pharmacogenes the likelihood of one individual carrying multiple variants in one pharmacogene is extremely high [19, 36], increasing the importance of haplotype phasing. Additionally, this high abundance of variants resulted in significantly larger haploblocks for the pharmacogenes compared to Gencode features.

Long read sequencing is comparable to short-read sequencing in regards to SNV detection and performs better in regards to haplotype phasing and complex SVs [19]. Haplotype phasing can potentially make the difference between an inferred intermediate metabolizer phenotype (two truncating variants on the same allele) and a poor metabolizer phenotype (two truncating variants on different alleles). Current PGx haplotyping strategies utilize computational phasing, leading to accurate phasing on a population scale but not always on an individual level. As drug adjustments are made on an individual level, accuracy in regards to phasing for one individual is crucial [37]. Here we have shown that long-read sequencing enables the majority of pharmacogenes to be fully phased into haploblocks without the need for pedigree data or for computational phasing.

Long-read sequencing also offers a full characterization of every variant in the selected PGx loci, including structural and rare variants, as indicated by the high precision and recall for SNVs, Indels and SVs. For example, the median read length (13.4kbp) is approximately three times larger than the size of the *CYP2D6* locus (4.4kbp), which allows for full characterization of the locus and potential CNVs. The large difference between DeepVariant and GATK for Indels can be explained by the use of long-read PacBio CCS data for the training of the DeepVariant caller. GATK was designed with the error mode of short read sequencing as a basis, with ~100 times more substitutions then indels. DeepVariant on the other hand has learned the error mode from the PacBio HiFi training data, which has a ratio of 30 times more indels compared to substitutions [19, 32]. Specifically, Indels and tandem repeat identification is significantly improved with the use of long reads and DeepVariant [19, 25]. This difference highlights once more the added benefit of long reads over short read sequencing in regard to the identification of complex variants.

For the studied individual, 1418 SNVs were identified in the selected clinical PGx loci (10 genes) of which 94% were fully phased, indicating a high abundance of variants in the pharmacogenes. Moreover, the phased nature of this data can help improve our understanding of haplotypes and variant combinations. Thus, long-read sequencing technologies have the potential of transforming our knowledge of genetic factors that play a role in variable drug response.

Prior to implementation of long-read sequencing into clinical practice, tools to assist the interpretation are needed. Several groups have made efforts to develop such translational tools for PGx [38–40]. However, there are still limitations to these tools. First, they often cover the entire range of known variants and their associated haplotypes. However, not for every *-haplotype the clinical impact is known, therefore this will occasionally result in haplotype of which the effect is unknown making it difficult to implement in clinical practice [34]. Secondly, the tools do not always provide the same result for the same individual [34], indicating that the assumptions on which these tools are based are not comparable. To only include clinically relevant *-haplotypes in our analysis, we have limited our analysis of the clinical utility to the panel of variants defined by the U-PGx consortium. It should be noted, however, that this does lead to the exclusion of the majority of variants in all PGx loci, due to the fact that there is not yet sufficient knowledge about the function of these variants.

To illustrate the impact of long reads on clinical PGx we have assessed the sequencing results in the context of the DPWG and CPIC guidelines. Based on the genetic variants observed in the studied individual, the guidelines recommended drug or dose adjustment for 22 drugs. Of all gene-drug interactions in the guidelines (53 for DPWG and 54 for CPIC), the vast majority (35 for both) was associated with a (partial) complex gene which could be fully resolved in a haploblock. As we have shown in this study, long-read sequencing is capable of resolving these complexities and constructing large haploblocks, allowing for more accurate haplotype calling. SNV panels and short-read sequencing, on the other hand, are capable of accurate variant identification but are limited in their ability to solve all complexities and in regard to haplotype phasing.

Nonetheless, it should be mentioned that not all pharmacogenes could be fully resolved. The key reason for this was the absence of heterozygous variants to allow for haploblock construction. This, in turn, leads to broken haploblocks and pharmacogenes which cannot be fully resolved. For the individual we studied this effect was apparent for *CYP2C19* and *DPYD* in particular. However, variant identification was still possible in the entire gene locus allowing for non-phased haplotype assignments. For these genes which could not be fully resolved, conventional haplotype approaches based on non-phased sequencing data can still be applied resulting in haplotype and phenotype predictions in line with current clinical practice. Moreover, for *DPYD* three of the four clinically relevant variants were still phased, two of which in the same haploblock. Indicating that a lack of complete phasing does not mean that none of the relevant variants can be phased. As the coverage was sufficient in all pharmacogenes, this lack of phasing is caused by the individuals genetic make-up, being a lack of heterozygous variants in this region, and not by the sequencing in itself, this is not easily resolved. For another individual the same problem of broken haploblocks might be observed in other genes depending on their genetics. While long-read sequencing for clinical pharmacogenomics seems promising, the costs and turn-around time associated with it are currently too high for potential high throughput PGx diagnostics [41]. Currently, this makes long-read sequencing not compatible with the quick SNV-arrays used in clinical PGx. However, sequencing costs are quickly decreasing. Moreover, pre-emptive genotyping becomes more popular which makes the longer turn-around time no longer an issue.

In this study, genetic data from a high-quality DNA sample was used. In clinical practice, high quality might not always be guaranteed. Nonetheless, previous applications of long-read sequencing in a clinical setting or with the use of clinically obtained DNA have resulted in good quality results [22–26]. Moreover, since 2020 a PacBio ultra-low DNA input workflow requiring only 5 ng of DNA has been available [42]. It is therefore expected that high quality sequencing results can be obtained with routinely collected clinical samples.

The accuracy and value of long-read sequencing has previously been investigated in whole genome data, which might make a targeted approach as we have presented here seem unnecessary [19]. However, it is well-established that the complexity of pharmacogenomic regions of the genome compromises the current assays in resolving their genetic makeup and thereby limiting the reliability and completeness of the phenotyping assays. The difference in genetic makeup of pharmacogenes compared to the general protein-coding genes makes the direct extrapolation from whole genome results unreliable. Most importantly, they contain more variants that together influence the drug response [19, 36, 43]. This high number of polymorphisms leads to the hypothesis that pharmacogenes can more easily be phased due to the higher abundance of heterozygous variants, as was confirmed in our study. Indeed, accuracy in the pharmacogenes was higher than that in other genes whereas short reads have a much lower accuracy in detecting genetic variants in these complex regions. The ability of long-read sequencing to resolve pharmacogenes was shown previously in targeted sequencing studies [7, 16, 26–30]. However, this study aimed at providing a comprehensive overview of the utility of long-read sequencing in resolving complex pharmacogenes and to inform on regions that remain challenging.

This study was limited to high quality data from a single subject and serves as a proof-of-concept for the application of long-read sequencing in PGx. Despite this limitation we feel that this is sufficient to serve as a proof-of-concept study investigating the potential of long-read sequencing for PGx. Based on these data regarding the variant calling accuracy and ability to resolve complex pharmacogene into phased haploblocks, we conclude that long-read sequencing data offers great opportunities to elucidate complex PGx loci and haplotype phasing while maintaining accurate variant calling in the selected pharmacogenes.

## Methods

### Data description

Publicly available long-read sequencing results of GIAB sample HG002 was sequenced with PacBio sequencing and analysed with the use of CCS (Circular Consensus Sequencing) reads, were obtained [19]. A GIAB sample was selected as these are extremely well characterised with benchmark results available [44]. CCS reads were generated using CCS software v.3.0.0 [19]. The obtained HiFi reads were aligned to GRCh38 reference genome using NGMLR aligner v0.2.7. Genetic variants were identified using GATK HaplotypeCaller (v.4.0.6.0) and DeepVariant (v.0.7.1). A set of 64 pharmacogenes that were previously described by Lauschke et al. [9] along with notoriously complex HLA-genes were selected for the PGx analysis (Table S1).

### Haploblock constructing

Variants called by GATK were phased using WhatsHap [45] to obtain phased SNV and Indel variants. From the phased reads, haploblocks were constructed and stored in GTF and BED files. Each haploblock was constructed by matching phased reads based on the variants they contain in order to increase the length of the sequence which can be resolved. One haploblock represents one stretch of unbroken sequence based on overlapping phased reads and stops when a region in the genome is covered only by reads without any variants, there is no longer a difference in variants between the two alleles or if the region lacks coverage.

Subsequently, all loci were categorised into one of three features: Gencode features (v28), PGx genes and intergenic features. Where a feature is defined as an annotated genomic region such as protein-coding genes, segmental duplicated regions, pseudogenes, etc. Gencode reference annotation for genetic features in the human genome (release 28) was used to investigate haploblock resolution of important loci such as protein coding genes. The Gencode project aims to classify and identify all gene features in human genomes including all annotations [46]. For each autosomal Gencode and PGx feature, the percentage of the feature that is covered in a haploblock is calculated (number of basepairs in haploblocks/total feature length). Regions with ≥90% haploblock coverage are classified as fully phased, whereas regions with no overlapping haploblocks remain unphased. All other regions are marked as partially phased.

Segmental duplications (SD) and repeat tracks are obtained from UCSC (University of California Santa Cruz) Genome Browser. Bedtools was used to identify overlapping regions between all tracks and annotations files discussed. For each locus, the percentage of segments overlapping with SDs or repeats is defined as ‘complex’.

### Clinical relevance

A previously developed pipeline was employed to assign haplotypes and phenotypes to clinically relevant pharmacogenes based on the DPWG guidelines [15]. The selected genes were based on the U-PGx consortium’s panel and consisted of 10 key pharmacogenes and 38 variants. The pipeline, which was originally designed for NGS data, did not include the *UGT1A1* and *HLA-B* genes which are present in the U-PGx consortium panel due to their complexities [15]. All genotypes are assessed based on their presence in the guidelines and on the number of drugs with an actionable advice, where actionable is defined as “a gene-drug interaction requiring a drug switch, dose adjustment or intensive monitoring”. For all pharmacogenes mentioned in the CPIC and DPWG guidelines, the number of gene-drug interactions are calculated.

### Recall and precision

To assess the accuracy of detecting different types of genetic variants in PGx genes, variant calling results were compared to the benchmark results from GIAB v.3.3.2 HG002 using the hap.py pipeline [47]. For SNVs and Indels, the benchmark v3.3. sequence is based on short-read sequencing [48]. Both the GATK variant caller (v.4.0.6.0) and DeepVariant (v.0.7.1) with the PacBio model were used to identify genetic variants. To assess recall and precision in complex regions, results were stratified using the stratifications from GIAB (https://github.com/genome-in-a-bottle/genome-stratifications). In addition, benchmarking results in GC-rich regions, homopolymers, tandem repeats, segmental duplications and UCSC repeat tracks were included in the analysis.

To assess the accuracy of SV calling in PGx genes, publicly available SV calls obtained with pbsv were downloaded from https://ftp-trace.ncbi.nlm.nih.gov/ReferenceSamples/giab/data/AshkenazimTrio/analysis/PacBio_pbsv_05212019/ and compared to the GIAB benchmark using truvari as previously described (https://github.com/PacificBiosciences/sv-benchmark) [19]. GIAB high confidence regions and SV callset were obtained from ftp://ftp-trace.ncbi.nlm.nih.gov/giab/ftp/data/AshkenazimTrio/analysis/ NIST_SVs_Integration_v0.6/. Since GIAB curation for SV is based on hg19, the genes were converted to the hg19 genome using the liftOver tool from UCSC. Bedtools was used to overlap pharmacogenes to high-confident regions from GIAB. The SV benchmark set only includes SV with a size larger than 50 bp, therefore the SV analysis is limited to SVs >50 bp.

## Supplementary information

supplementary file legends

Table S1

Table S2

Table S3

Table S4

Table S5

Table S6

Figure S1

Figure S2

Figure S3

Figure S4

## Data Availability

The code developed to generate the results in this study is available upon request.
