# Supplementary material for: Application of long-read sequencing to elucidate complex pharmacogenomic regions: a proof of principle
Source: Pharmacogenomics J. 2021 Nov 5;22(1):75–81. doi: 10.1038/s41397-021-00259-z (PMC8794781; doi:10.1038/s41397-021-00259-z)
Supplement: Supplementary file 1 — supplementary file legends [file 41397_2021_259_MOESM1_ESM.docx]

### Supplementary files

**Figure S1: Recall and precision stratified by GC-content**

**Figure S2: Haploblock resolution of GENCODE features.** For each of the most

2 common features, the percentage to be resolved into haploblocks compared to the feature length. The red line reflects the mean read length.

**Figure S3: proportion of features which were covered and fully phased into haploblocks**. The majority of each Genomic feature type was completely resolved in overlapping

haploblocks.

**Figure S4: Alignment and phasing of reads for *CYP2D6* (A), *VKORC1* (B), *DPYD* (C), *CYP2C19* (D), *CYP2B6* (E) and the *CYP3A* locus (F).** Allele 1 and 2 indicate the phased reads. Reads in allele X could not be mapped to either one of the alleles. Reads were aligned to GRCh38.

**Table S1: Selected pharmacogenes.** 100 pharmacogenes were selected based on their complexity. Genes were extracted from Lauschke et al in addition to the notoriously complex HLA-genes.

**Table S2: Stratified benchmarking results.** Benchmarking results were stratified to reflect the accuracy in different complex regions. Both DeepVariant and GATK variant caller were compared

**Table S3: overlap between the high confidence GIAB data (HG002_SVs_Tier1_v0.6.bed) and the PGx genes**

**Table S4: Structural variants identified in the pharmacogenes**

**Table S5: Clinical pharmacogenomics results**. The U-PGx consortium’s variant panel was used to assign clinical genotypes and phenotypes. 38 variants in 10 genes were selected. Results from the sequencing data of the human in a bottle sample are included. NM: normal metabolizer, IM: intermediate metabolizer, PM: poor metabolizer, GAS: gene activity score in bold: pharmacogenomic mutations compared to the reference.

**Table S6: Complexity and haploblocks of studied genes included in the CPIC and DPWG guidelines.** Actionable is here defined as, a guideline which recommends a dose change or drug switch. CPIC: Clinical Pharmacogenetics Implementation Consortium, DPWG: Dutch Pharmacogenetics Working group.
