# Supplementary material for: Application of long-read sequencing to elucidate complex pharmacogenomic regions: a proof of principle
Source: Pharmacogenomics J. 2021 Nov 5;22(1):75–81. doi: 10.1038/s41397-021-00259-z (PMC8794781; doi:10.1038/s41397-021-00259-z)
Supplement: Supplementary file 2 — Table S1 [file 41397_2021_259_MOESM2_ESM.docx]

**Supplementary table 1: selected pharmacogenes.** 100 pharmacogenes were selected based on their complexity. Genes were extracted from Lauschke et al in addition to the notoriously complex HLA-genes.

| ABCB1 | CYP4F2 | HLA-DQB3 | NAT1 |
| --- | --- | --- | --- |
| ABCC2 | DPYD | HLA-DRA | NAT2 |
| ABCG2 | DRD2 | HLA-DRB1 | NQO1 |
| ACE | EGFR | HLA-DRB5 | NR1I2 |
| ADH1A | F5 | HLA-DRB6 | P2RY1 |
| ADH1B | G6PD | HLA-DRB9 | P2RY12 |
| ADH1C | GSTM1 | HLA-E | PTGS2 |
| ADRB1 | GSTP1 | HLA-F | RYR1 |
| ADRB2 | GSTT2 | HLA-G | SCN5A |
| AHR | HLA-A | HLA-H | SLC15A2 |
| ALDH1A1 | HLA-B | HLA-J | SLC19A1 |
| ALOX5 | HLA-C | HLA-K | SLC22A1 |
| CFTR | HLA-DMA | HLA-L | SLC22A2 |
| COMT | HLA-DMB | HLA-N | SLC22A6 |
| CYP1A1 | HLA-DOA | HLA-P | SLCO1B1 |
| CYP1A2 | HLA-DOB | HLA-S | SLCO1B3 |
| CYP2A6 | HLA-DPA1 | HLA-T | SULT1A1 |
| CYP2B6 | HLA-DPA2 | HLA-U | TPMT |
| CYP2C19 | HLA-DPA3 | HLA-V | TYMS |
| CYP2C8 | HLA-DPB1 | HLA-W | UGT1A1 |
| CYP2C9 | HLA-DPB2 | HLA-Z | UGT2B15 |
| CYP2D6 | HLA-DQA1 | HMGCR | UGT2B17 |
| CYP2E1 | HLA-DQA2 | KCNH2 | UGT2B7 |
| CYP3A4 | HLA-DQB1 | KCNJ11 | VDR |
| CYP3A5 | HLA-DQB2 | MTHFR | VKORC1 |
