# Supplementary material for: Application of long-read sequencing to elucidate complex pharmacogenomic regions: a proof of principle
Source: Pharmacogenomics J. 2021 Nov 5;22(1):75–81. doi: 10.1038/s41397-021-00259-z (PMC8794781; doi:10.1038/s41397-021-00259-z)
Supplement: Supplementary file 3 — Table S2 [file 41397_2021_259_MOESM3_ESM.docx]

**Supplementary table 2. Stratified benchmarking results.** Benchmarking results were stratified to reflect the accuracy in different complex regions. Both DeepVariant and GATK variant caller were compared

| **Stratified region** | **Variant caller** | **SNVs** | | | **Indels** | | |
| --- | --- | --- | --- | --- | --- | --- | --- |
|  |  | **Precision (%)** | **Recall (%)** | **F1 (%)** | **Precision (%)** | **Recall(%)** | **F1 (%)** |
| Segmental duplications | GATK haplotype caller | 100 | 88.9 | 94.1 | 100.0 | 88.9 | 94.1 |
|  | DeepVariant  (CCS model) | 100 | 100 | 100 | 100 | 100 | 100 |
| Functional regions (refseq cds)* | GATK haplotype caller | 100 | 100 | 100 | NA | NA | NA |
|  | DeepVariant  (CCS model) | 100 | 100 | 100 | NA | NA | NA |
| Repeats** | GATK haplotype caller | 99.8 | 100.0 | 99.9 | 94.1 | 83.5 | 88.5 |
|  | DeepVariant  (CCS model) | 99.8 | 100.0 | 100.0 | 98.4 | 97.6 | 98.0 |
| All tandem repeats and homopolymers | GATK haplotype caller | 98.9 | 99.6 | 99.3 | 91.5 | 79.2 | 84.9 |
|  | DeepVariant  (CCS model) | 99.6 | 100.0 | 99.8 | 98.1 | 97.4 | 97.8 |

*Refseq CDS: no indels in the truth set
**Repeats: repeat mask UCSC
SNV: Single Nucleotide Variant, GATK: Genome Analysis Toolkit, CCS: Circular Consensus.
