# Supplementary material for: Application of long-read sequencing to elucidate complex pharmacogenomic regions: a proof of principle
Source: Pharmacogenomics J. 2021 Nov 5;22(1):75–81. doi: 10.1038/s41397-021-00259-z (PMC8794781; doi:10.1038/s41397-021-00259-z)
Supplement: Supplementary file 5 — Table S4 [file 41397_2021_259_MOESM5_ESM.docx]

**Supplementary table 4. Structural variants identified in the pharmacogenes.**

| **Benchmark agreement** | **Chromosome** | **position (GRCh37)** | **Gene** | **ID** | **Type** | **Length** |
| --- | --- | --- | --- | --- | --- | --- |
| True positive | 1 | 97717628 | *DPYD* | pbsv.INS.1687 | INS | 329 |
|  | 1 | 169523397 | *F5* | pbsv.INS.2409 | INS | 96 |
|  | 3 | 151086421 | *P2RY12* | pbsv.DEL.9734 | DEL | 65 |
|  | 4 | 69959841 | *UGT2B7* | pbsv.DEL.11945 | DEL | 324 |
|  | 4 | 89037668 | *ABCG2* | pbsv.DEL.12237 | DEL | 336 |
|  | 6 | 160546587 | *SLC22A1* | pbsv.INS.19451 | INS | 82 |
|  | 6 | 160640128 | *SLC22A2* | pbsv.INS.19452 | INS | 3402 |
|  | 6 | 160656527 | *SCL22A2* | pbsv.INS.19453 | INS | 351 |
|  | 7 | 17094700 | *AHR* | pbsv.INS.20366 | INS | 485 |
|  | 7 | 99273261 | *CYP3A5* | pbsv.INS.21674 | INS | 185 |
|  | 9 | 75542969 | *ALDH1A1* | pbsv.INS.26157 | INS | 349 |
|  | 9 | 75669517 | *ALDH1A1* | pbsv.DEL.26148 | DEL | 279 |
|  | 10 | 96809614 | *CYP2C8* | pbsv.DEL.29091 | DEL | 89 |
|  | 10 | 101587737 | *ABCC2* | pbsv.INS.29155 | INS | 218 |
|  | 12 | 48334643 | *VDR* | pbsv.INS.33113 | INS | 391 |
|  | 19 | 15993273 | *CYP4F2* | pbsv.DEL.44752 | DEL | 118 |
|  | 19 | 39032094 | *RYR1* | pbsv.INS.45199 | INS | 135 |
|  | 19 | 39037384 | *RYR1* | pbsv.INS.45200 | INS | 914 |
|  | 19 | 39044965 | *RYR1* | pbsv.INS.45201 | INS | 889 |
|  | 21 | 46951123 | *SLC19A1* | pbsv.INS.47966 | INS | 660 |
|  | 22 | 19947322 | *COMT* | pbsv.DEL.48142 | DEL | 67 |
| False positive | 19 | 39031163 | *RYR1* | pbsv.DEL.45187 | DEL | 58 |
| False negative | 3 | 38626083 | *SCN5A* | HG2_PB_pbsv_3189 | INS | 6312 |
|  | 4 | 89092659 | *ABCG2* | HG4_PB_HySA_7022 | INS | 4779 |
