# Supplementary material for: Application of long-read sequencing to elucidate complex pharmacogenomic regions: a proof of principle
Source: Pharmacogenomics J. 2021 Nov 5;22(1):75–81. doi: 10.1038/s41397-021-00259-z (PMC8794781; doi:10.1038/s41397-021-00259-z)
Supplement: Supplementary file 6 — Table S5 [file 41397_2021_259_MOESM6_ESM.docx]

**Supplementary table 5: clinical pharmacogenomics results.** The U-PGx consortium’s variant panel was used to assign clinical genotypes and phenotypes. 38 variants in 10 genes were selected. Results from the sequencing data of the human in a bottle sample are included. NM: normal metabolizer, IM: intermediate metabolizer, PM: poor metabolizer, GAS: gene activity score in bold: pharmacogenomic mutations compared to the reference.

| **Gene** | **diplotype** | **Predicted phenotype** | **RS-number** | **GRch38 annotation** | ***-allele** | **Genotype** |
| --- | --- | --- | --- | --- | --- | --- |
| CYP2B6 | *1/*1 | NM | rs2279343 | NC_000019.10:g.41009358A>G | *4 | AA |
|  |  |  | rs28399499 | NC_000019.10:g.41012316T>C | *18 | TT |
|  |  |  | rs3745274 | NC_000019.10:g.41006936G>T | *9 | GG |
|  |  |  | rs2279343; rs28399499 | NC_000019.10:g.41009358A>G; NC_000019.10:g.41012316T>C | *16 | AA;TT |
|  |  |  | rs2279343; rs3745274 | NC_000019.10:g.41009358A>G; NC_000019.10:g.41006936G>T | *6 | AA;GG |
| CYP2C19 | *1/*1 | NM | rs12248560 | NC_000010.11:g.94761900C>T | *17 | CC |
|  |  |  | rs17884712 | NC_000010.11:g.94775489G>A | *9 | GG |
|  |  |  | rs28399504 | NC_000010.11:g.94762706A>G | *4A/B | AA |
|  |  |  | rs41291556 | NC_000010.11:g.94775416T>C | *8 | TT |
|  |  |  | rs4244285 | NC_000010.11:g.94781859G>A | *2 | GG |
|  |  |  | rs4986893 | NC_000010.11:g.94780653G>A | *3 | GG |
|  |  |  | rs56337013 | NC_000010.11:g.94852738C>T | *5 | CC |
|  |  |  | rs6413438 | NC_000010.11:g.94781858C>T | *10 | CC |
|  |  |  | rs72552267 | NC_000010.11:g.94775453G>A | *6 | GG |
| CYP2C9 | *1/*1 | NM | rs1057910 | NC_000010.11:g.94981296A>C | *3 | AA |
|  |  |  | rs1799853 | NC_000010.11:g.94942290C>T | *2 | CC |
|  |  |  | rs28371685 | NC_000010.11:g.94981224C>T | *11 | CC |
|  |  |  | rs28371686 | NC_000010.11:g.94981301C>G | *5 | CC |
| CYP2D6 | *1/*4 | IM | rs1065852 | NC_000022.11:g.42130692G>A | *10 | **GA** |
|  |  |  | rs28371706 | NC_000022.11:g.42129770G>A | *17 | GG |
|  |  |  | rs28371725 | NC_000022.11:g.42127803C>T | *41 | CC |
|  |  |  | rs35742686 | NC_000022.11:g.42128242del | *3 | TT |
|  |  |  | rs3892097 | NC_000022.11:g.42128945C>T | *4 | **CT** |
|  |  |  | rs3892097; rs1065852 | NC_000022.11:g.42128945C>T; NC_000022.11:g.42130692G>A | *4 | **CT;GA** |
|  |  |  | rs5030655 | NC_000022.11:g.42129084del | *6 | AA |
|  |  |  | rs5030656 | NC_000022.11:g.42128176_42128178del | *9 | TCT/TCT |
|  |  |  | rs5030865 | NC_000022.11:g.42129033C>A | *8 | CC |
|  |  |  | rs5030865 | NC_000022.11:g.42129033C>T | *14B | CC |
|  |  |  | rs5030865; rs1065852 | NC_000022.11:g.42129033C>T; NC_000022.11:g.42130692G>A | *14A | CC;GG |
| CYP3A5 | *3/*3 | PM | rs10264272 | NC_000007.14:g.99665212C>T | *6 | CC |
|  |  |  | rs41303343 | NC_000007.14:g.99652771insA | *7 | -/- |
|  |  |  | rs776746 | NC_000007.14:g.99672916C>T | *3 | **TT** |
| DPYD | *1/*1 | GAS:2 | rs3918290 | NC_000001.11:g.97450058C>T | *2A | CC |
|  |  |  | rs55886062 | NC_000001.11:g.97515787A>C | *13 | AA |
|  |  |  | rs56038477 | NC_000001.11:g.97573863C>T | 1236G>A | CC |
|  |  |  | rs67376798 | NC_000001.11:g.97082391T>A | 2846A>T | TT |
| F5L | FVL negative | FVL negative | rs6025 | NC_000001.11:g.169549811C>T | FVL positive | CC |
| SLCO1B1 | *1/*1 | 521TT | rs4149056 | NC_000012.12:g.21178615T>C | *5 | TT |
| TPMT | *1/*1 | NM | rs1142345; rs1800460 | NC_000006.12:g.18130687T>C; NC_000006.12:g.18138997C>T | *3A | TT;CC |
|  |  |  | rs1142345 | NC_000006.12:g.18130687T>C | *3C | TT |
|  |  |  | rs1800460 | NC_000006.12:g.18138997C>T | *3B | CC |
|  |  |  | rs1800462 | NC_000006.12:g.18143724C>G | *2 | CC |
| VKORC1 | 1173TT | 1173TT | rs9934438 | NC_000016.10:g.31093557G>A | 1173C>T | **AA** |
