# Supplementary material for: Application of long-read sequencing to elucidate complex pharmacogenomic regions: a proof of principle
Source: Pharmacogenomics J. 2021 Nov 5;22(1):75–81. doi: 10.1038/s41397-021-00259-z (PMC8794781; doi:10.1038/s41397-021-00259-z)
Supplement: Supplementary file 7 — Table S6 [file 41397_2021_259_MOESM7_ESM.docx]

**Supplementary table 6:** **Complexity and haploblocks of studied genes included in the CPIC and DPWG guidelines.** Actionable is here defined as, a guideline which recommends a dose change or drug switch. CPIC: Clinical Pharmacogenetics Implementation Consortium, DPWG: Dutch Pharmacogenetics Working group.

| **Gene** | **Percentage complex (%)** | **Percentage resolved in haploblocks (%)** | **Number of actionable drugs in DPWG** | **Number of actionable drugs in CPIC** |
| --- | --- | --- | --- | --- |
| CFTR | 42,2 | 92,5 | - | 1 |
| COMT | 51,2 | 92,8 | 0 | - |
| CYP1A2 | 33,6 | 100,0 | 0 | - |
| CYP2B6 | 100,0 | 100,0 | 1 | 1 |
| CYP2C19 | 83,6 | 33,8 | 10 | 15 |
| CYP2C9 | 72,0 | 38,8 | 2 | 10 |
| CYP2D6 | 100,0 | 100,0 | 21 | 14 |
| CYP3A4 | 100,0 | 92,6 | 0 | - |
| CYP3A5 | 49,4 | 66,6 | 1 | 1 |
| CYP4F2 | 51,4 | 100,0 | - | 1 |
| DPYD | 40,0 | 55,2 | 4 | 2 |
| F5 | 41,9 | 100,0 | 1 | - |
| G6PD | 36,4 | 0,0 | - | 1 |
| HLA-A | 100,0 | 100,0 | 1 | 2 |
| HLA-B | 62,1 | 100,0 | 7 | 6 |
| RYR1 | 53,4 | 100,0 | - | 7 |
| SLCO1B1 | 69,6 | 54,2 | 2 | 1 |
| TPMT | 52,3 | 100,0 | 3 | 3 |
| UGT1A1 | 35,4 | 100,0 | 1 | 1 |
| VKORC1 | 40,3 | 71,1 | 3 | 1 |
