# Supplementary figures and images for: Application of long-read sequencing to elucidate complex pharmacogenomic regions: a proof of principle
Source: Pharmacogenomics J. 2021 Nov 5;22(1):75–81. doi: 10.1038/s41397-021-00259-z (PMC8794781; doi:10.1038/s41397-021-00259-z)

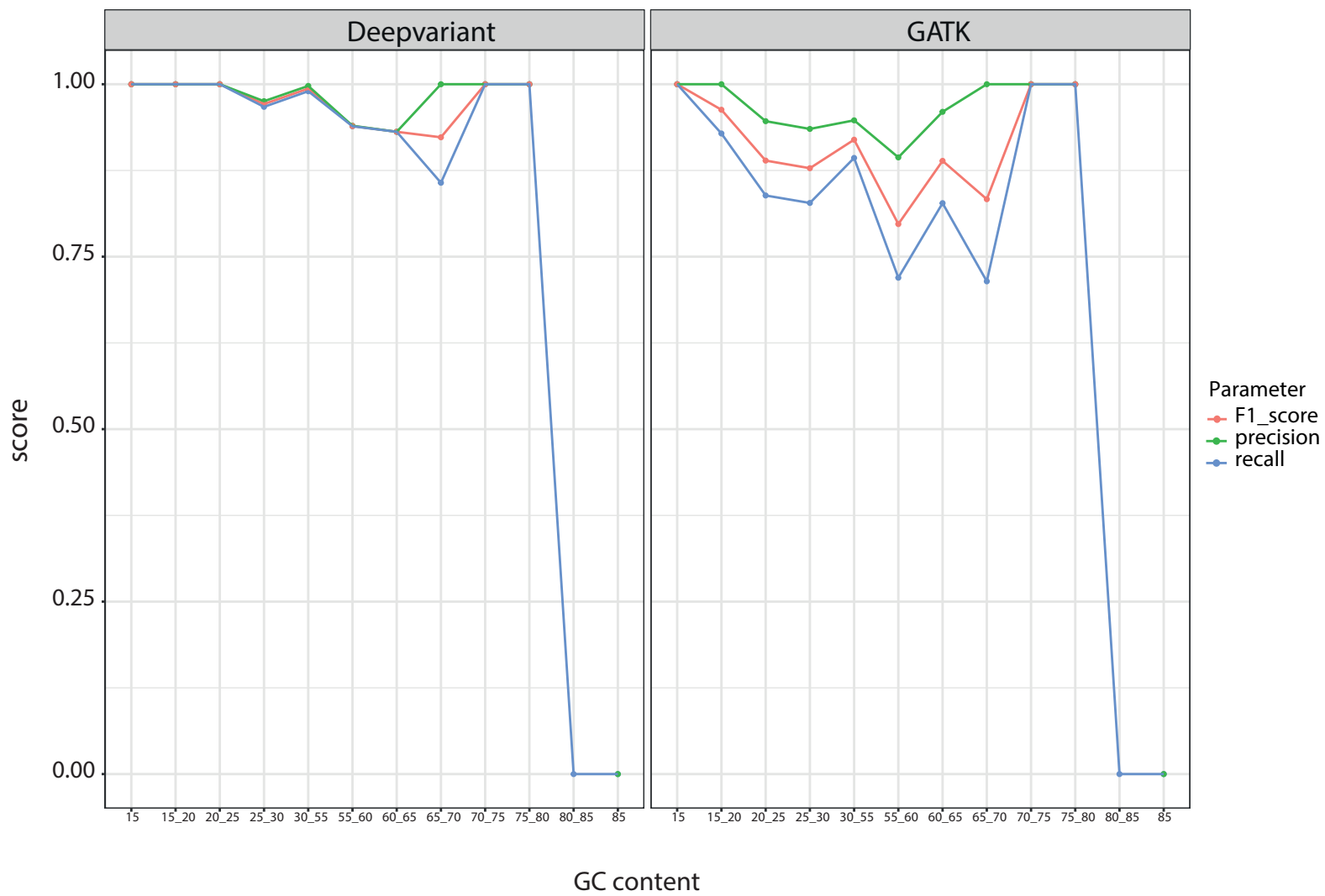

**Supplementary figure 1. Recall and precision stratified by GC content.**

Supplement: Supplementary file 8 — Figure S1 [file 41397_2021_259_MOESM8_ESM.pdf]
