# Supplementary material for: Application of long-read sequencing to elucidate complex pharmacogenomic regions: a proof of principle
Source: Pharmacogenomics J. 2021 Nov 5;22(1):75–81. doi: 10.1038/s41397-021-00259-z (PMC8794781; doi:10.1038/s41397-021-00259-z)
Supplement: Supplementary file 9 — Figure S2 [file 41397_2021_259_MOESM9_ESM.pdf]

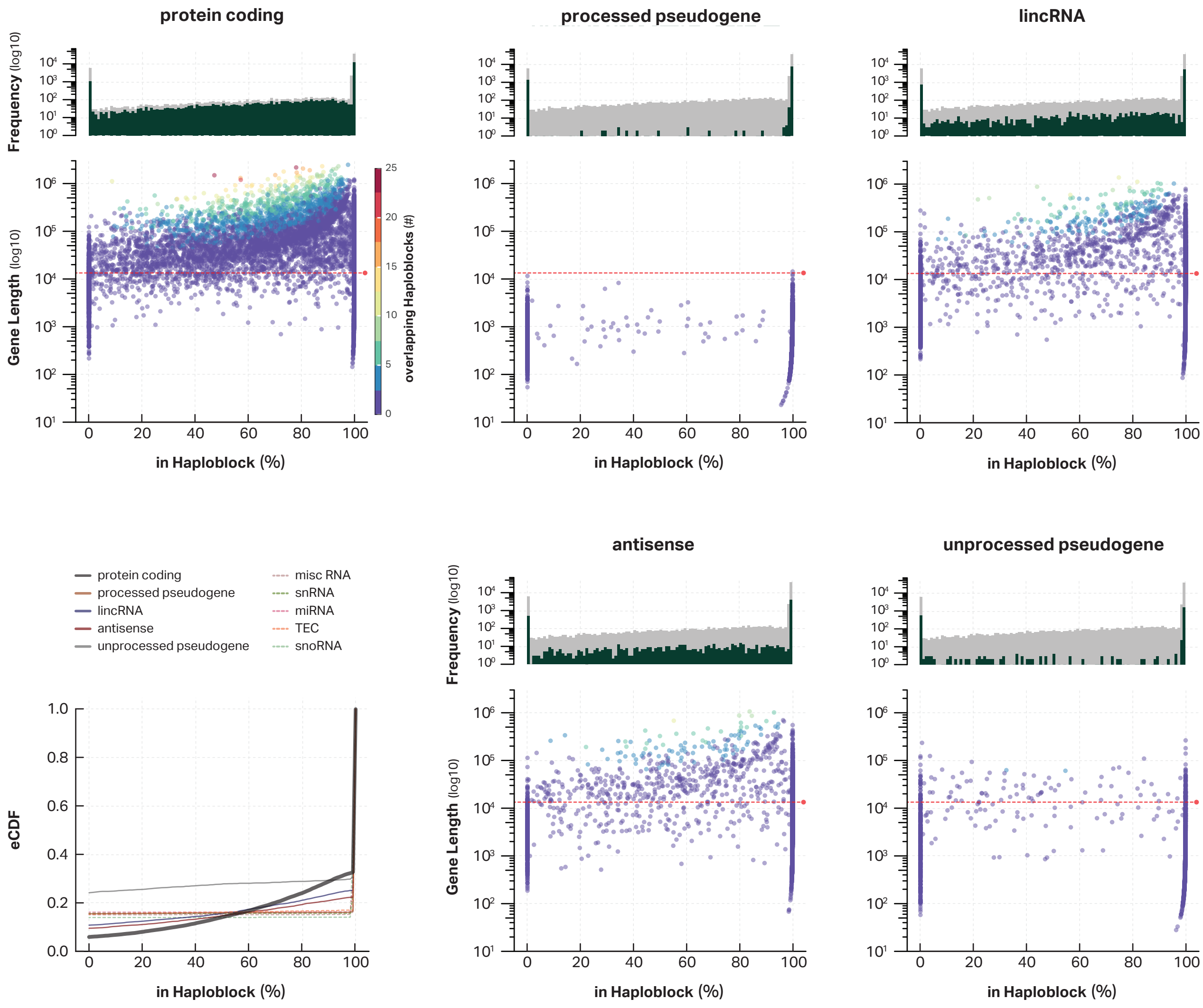

Supplementary figure 2 - Haploblock resolution of GENCODE features. For each of the most common features, the percentage to be resolved into haploblocks compared to the feature length. The red line reflects the mean read length.
