# Supplementary material for: Application of long-read sequencing to elucidate complex pharmacogenomic regions: a proof of principle
Source: Pharmacogenomics J. 2021 Nov 5;22(1):75–81. doi: 10.1038/s41397-021-00259-z (PMC8794781; doi:10.1038/s41397-021-00259-z)
Supplement: Supplementary file 10 — Figure S3 [file 41397_2021_259_MOESM10_ESM.pdf]

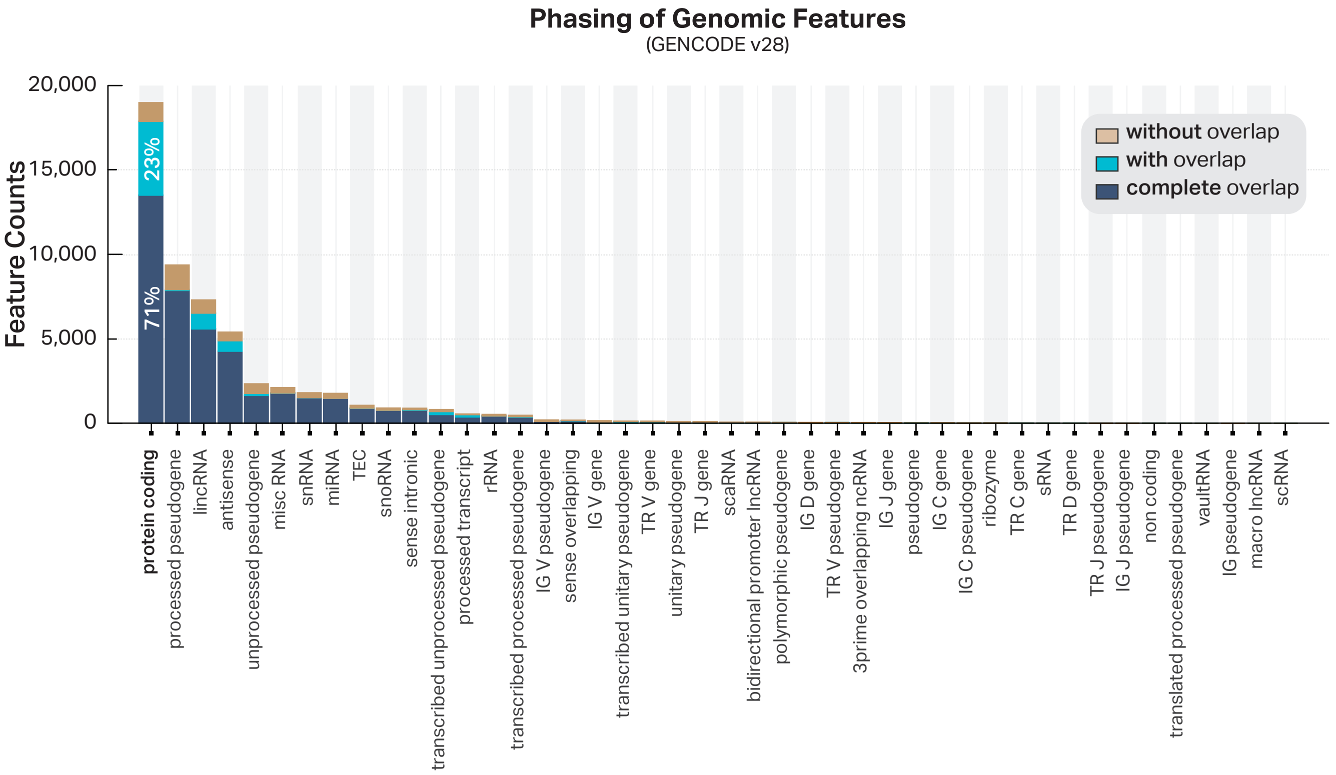

Supplementary figure 3: proportion of features which were covered and fully phased into haploblocks. The majority of each Genomic feature type was completely resolved in overlapping haploblocks.
