# Supplementary material for: Application of long-read sequencing to elucidate complex pharmacogenomic regions: a proof of principle
Source: Pharmacogenomics J. 2021 Nov 5;22(1):75–81. doi: 10.1038/s41397-021-00259-z (PMC8794781; doi:10.1038/s41397-021-00259-z)
Supplement: Supplementary file 11 — Figure S4 [file 41397_2021_259_MOESM11_ESM.pdf]

Supplementary figure 4: Alignment and phasing of reads for *CYP2D6* (A), *VKORC1* (B), *DPYD* (C), *CYP2C19* (D), *CYP2B6* (E) and the *CYP3A* locus (F). Allele 1 and 2 indicate the phased reads. Reads in allele X could not be mapped to either one of the alleles. Reads were aligned to GRCh38.

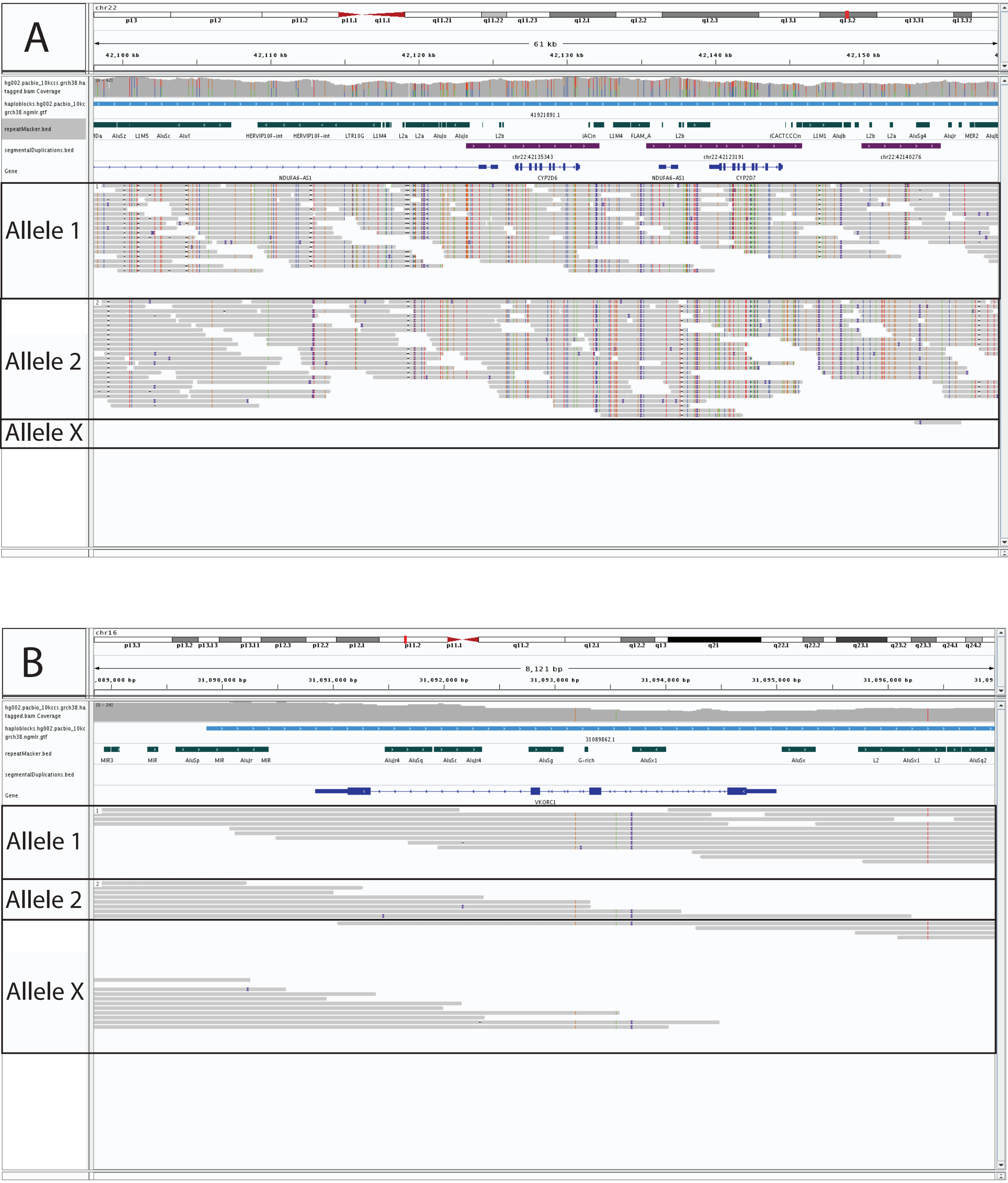

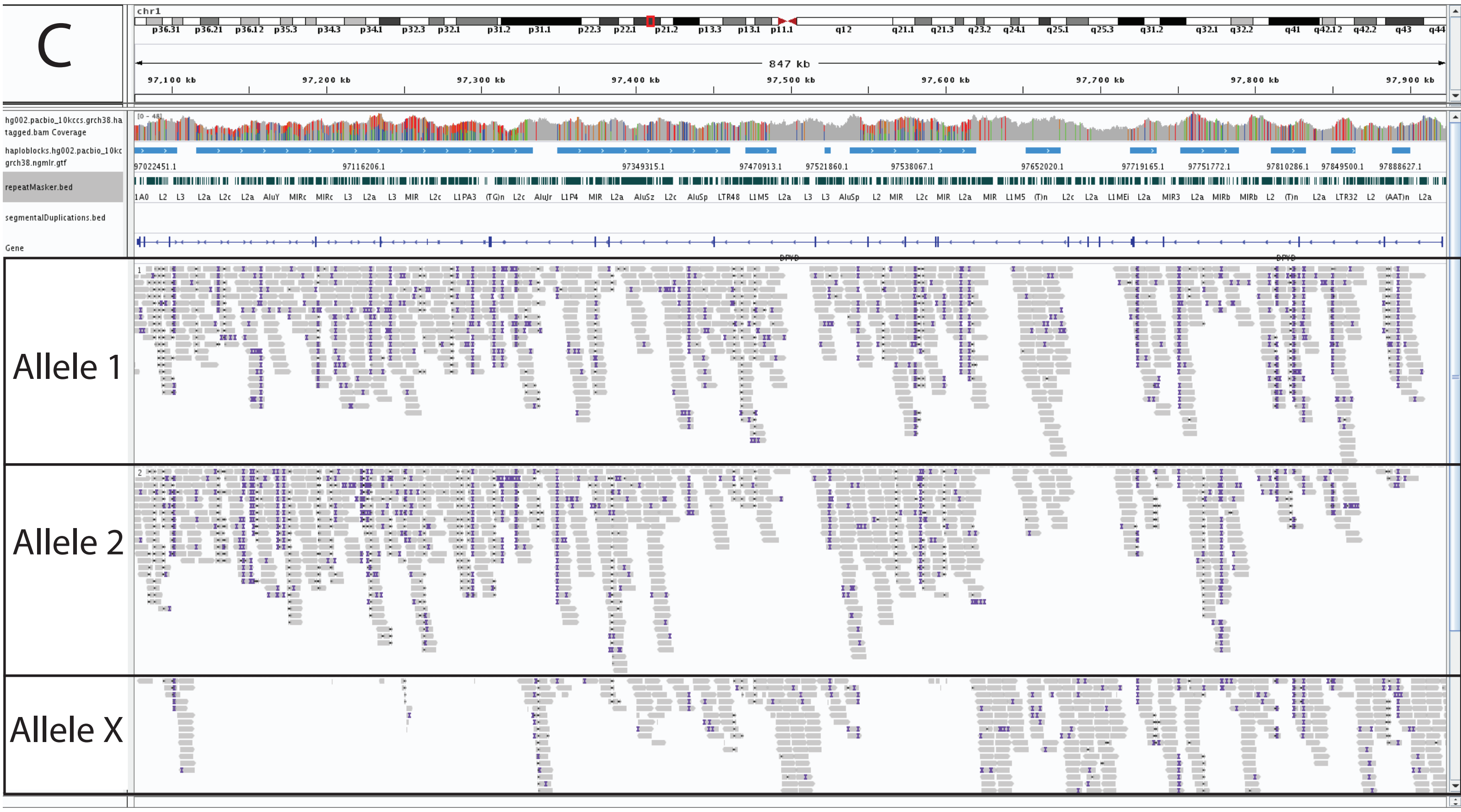
C
chr1
847 kb
97,100 kb
97,200 kb
97,300 kb
97,400 kb
97,500 kb
97,600 kb
97,700 kb
97,800 kb
97,900 kb
hg002.pacbio_10kccs.grch38.ha tagged.bam Coverage
haploblocks.hg002.pacbio_10kc grch38.ngmlr.gtf
repeatMasker.bed
segmentalDuplications.bed
Gene
DPYD
Allele 1
Allele 2
Allele X

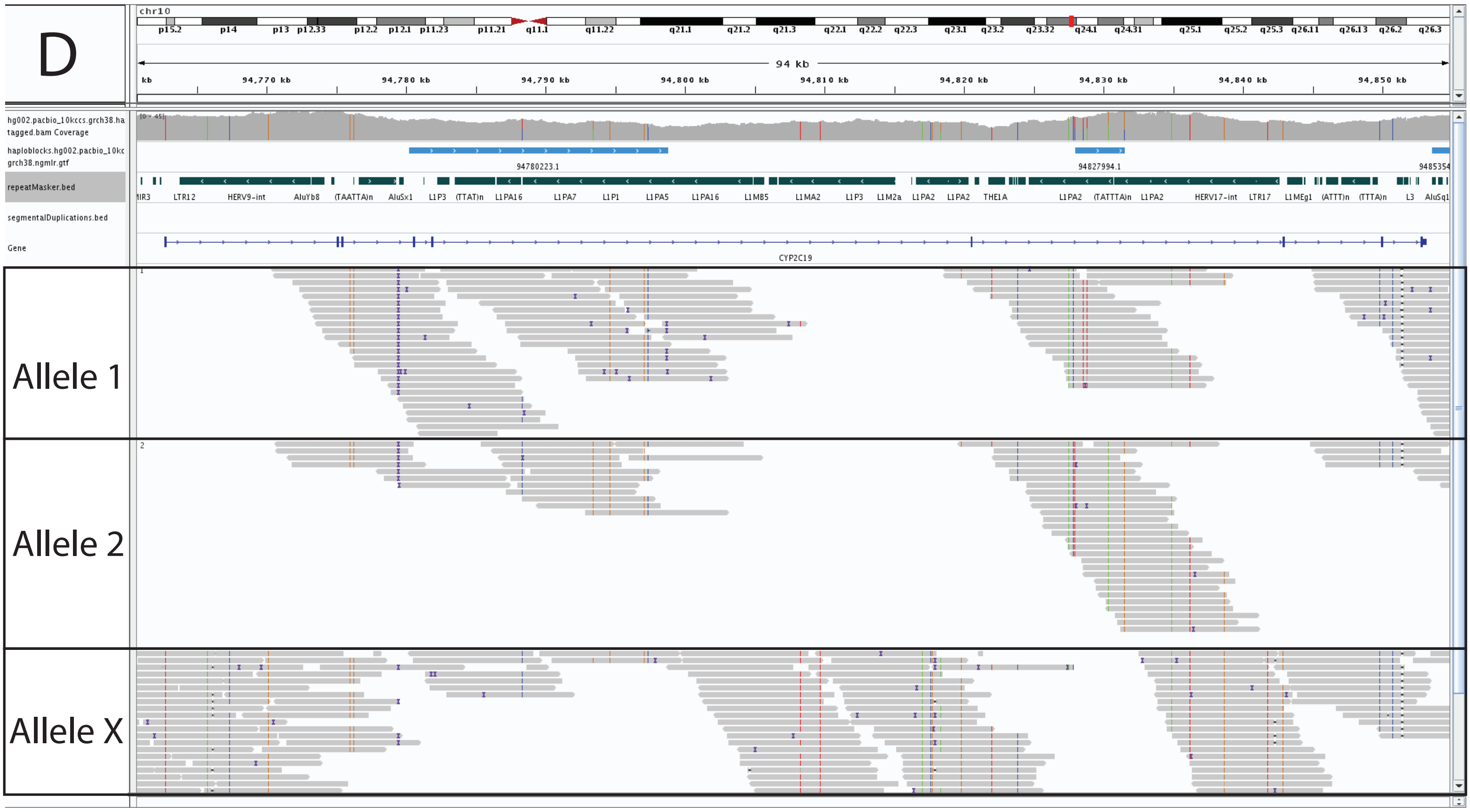
D
chr10
94 kb
94,770 kb
94,780 kb
94,790 kb
94,800 kb
94,810 kb
94,820 kb
94,830 kb
94,840 kb
94,850 kb
hg002.pacbio_10kccs.grch38.ha tagged.bam Coverage
haploblocks.hg002.pacbio_10kc grch38.ngmlr.gtf
repeatMasker.bed
segmentalDuplications.bed
Gene
CYP2C19
Allele 1
Allele 2
Allele X

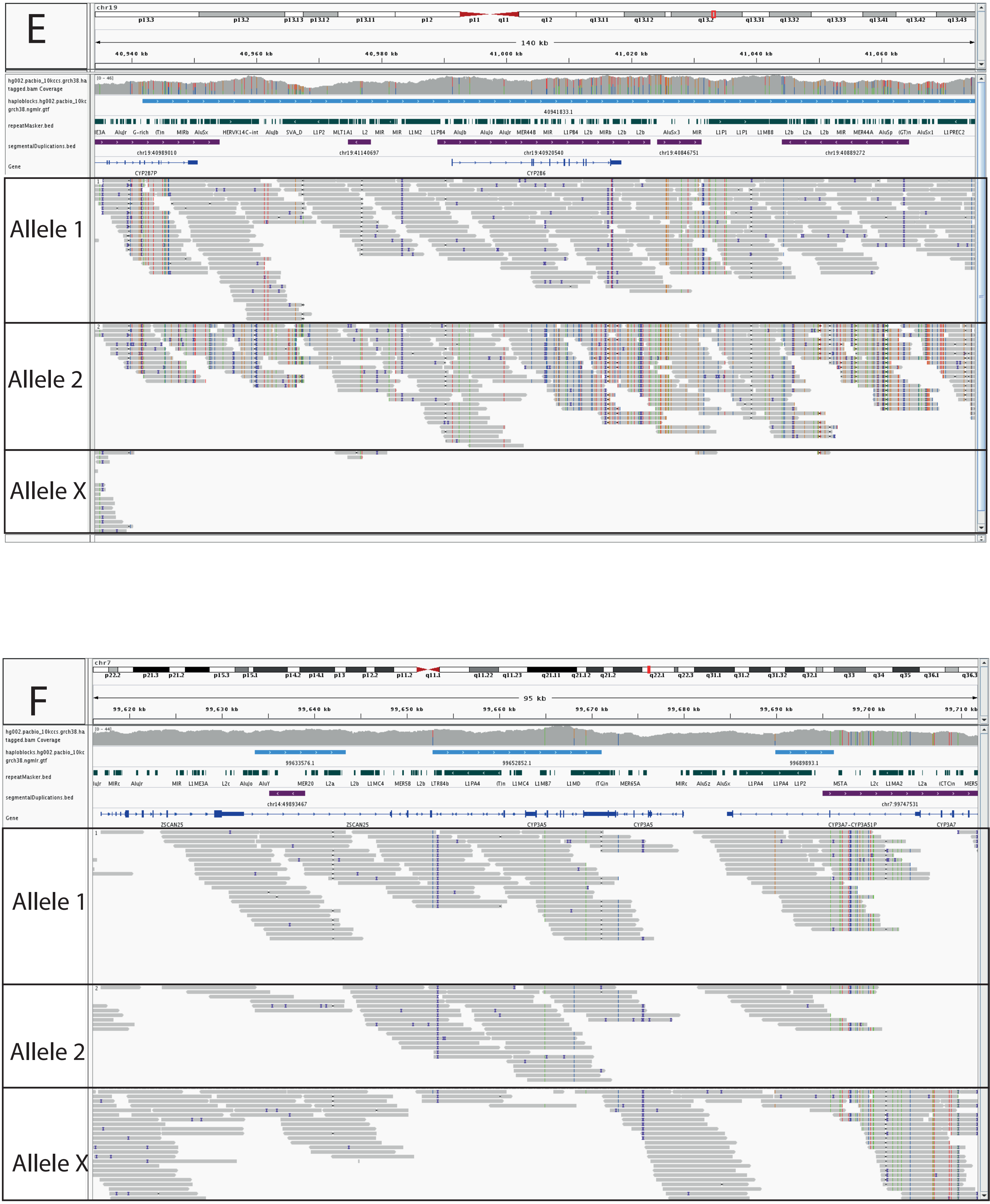
E
chr19
140 kb
40,940 kb
40,960 kb
40,980 kb
41,000 kb
41,020 kb
41,040 kb
41,060 kb
hg002.pacbio_10kccs.grch38.ha tagged.bam Coverage
haploblocks.hg002.pacbio_10kc grch38.ngmlr.gtf
repeatMasker.bed
segmentalDuplications.bed
Gene
40941833.1
chr19:40989010
chr19:41140697
chr19:40920540
chr19:40846751
chr19:40889272
CYP2B7P
CYP2B6
Allele 1
Allele 2
Allele X
F
chr7
95 kb
99,620 kb
99,630 kb
99,640 kb
99,650 kb
99,660 kb
99,670 kb
99,680 kb
99,690 kb
99,700 kb
99,710 kb
hg002.pacbio_10kccs.grch38.ha tagged.bam Coverage
haploblocks.hg002.pacbio_10kc grch38.ngmlr.gtf
repeatMasker.bed
segmentalDuplications.bed
Gene
99633576.1
99652852.1
99689893.1
chr14:49893467
chr7:99747531
ZSCAN25
ZSCAN25
CYP3A5
CYP3A5
CYP3A7-CYP3A51P
CYP3A7
Allele 1
Allele 2
Allele X
